# Supplementary material for: Bioinformatics of Differentially Expressed Genes in Phorbol 12-Myristate 13-Acetate-Induced Megakaryocytic Differentiation of K562 Cells by Microarray Analysis
Source: Int J Mol Sci. 2022 Apr 11;23(8):4221. doi: 10.3390/ijms23084221 (PMC9031040; doi:10.3390/ijms23084221)
Supplement: Supplementary file 1 [file ijms-23-04221-s001.zip › ijms-1650015_Supplementary materials _R.pdf]

# Bioinformatics of differentially expressed genes in phorbol 12-myristate 13-acetate-induced megakaryocytic differentiation of K562 cells by microarray analysis

**Seung-Hoon Lee [1,2,3], Na Rae Park [1,3] and Jung-Eun Kim [1,2,3,*]**

* Correspondence: kjeun@knu.ac.kr

## Supplementary Materials

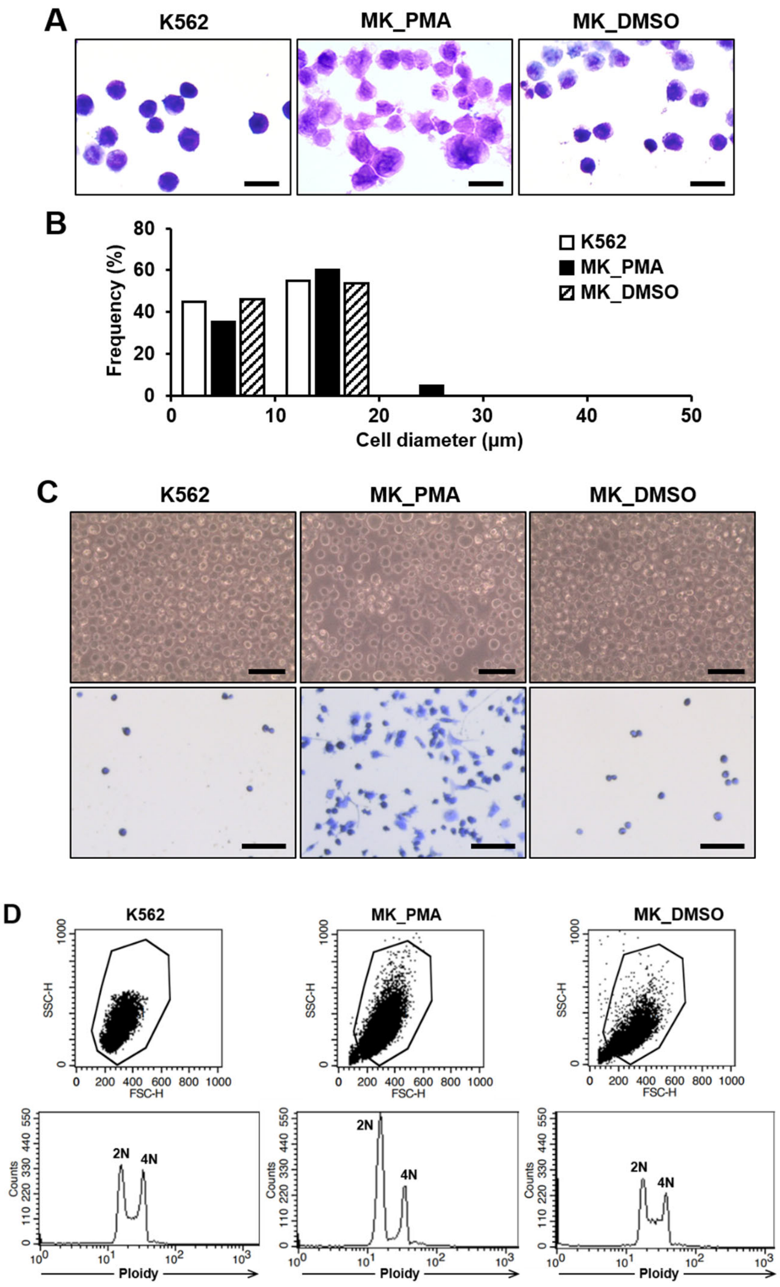


**Figure S1.** Verification of K562 cell differentiation into megakaryocytes on day 5 of differentiation. (**A,B**) The morphology and diameter of K562 cells (K562), PMA-induced megakaryocytes (MK_PMA), and DMSO-treated megakaryocytes (MK_DMSO) were comparatively analyzed by cytological staining (A) and size quantification (B). K562 cells were treated with PMA or DMSO for 5 days. Cell size was classified into 10-μm increments ranging from 0 to 50 μm in diameter. A total of 396 K562, 422 MK_PMA, and 418 MK_DMSO were quantified by the percentage of frequency using the i-Solution image analysis program. Scale bar = 20 μm. (**C**) Verification of K562 cell differentiation into megakaryocytes by cell adherence. Adhesive properties to the culture plate were verified before and after Hemacolor Rapid staining, as shown in the upper and lower panels, respectively. Scale bar = 100 μm. (**D**) Representative data showing ploidy of megakaryocytes after 5 days of differentiation with PMA. MK_PMA were compared to K562 or MK_DMSO.

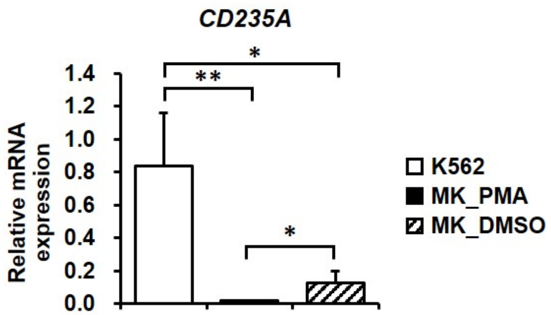


**Figure S2.** Verification of K562 cell differentiation into megakaryocytes by CD235a expression. The primers used to amplify *CD235A* mRNA expression by qPCR were 5'-CAA ACG GGA CAC ATA TGC AG-3' and 5'-TCC AAT AAC ACC AGC CAT CA-3'. The relative mRNA expression level was represented as mean ± standard deviation of three independent experiments. MK, megakaryocytes; *, $p < 0.05$ and **, $p < 0.01$

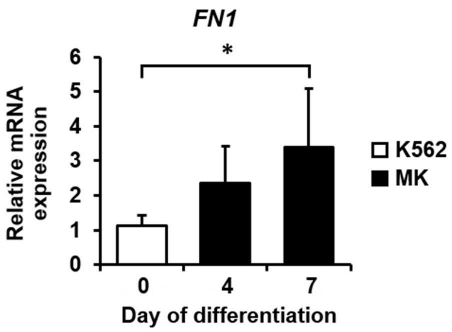


**Figure S4.** *FN1* expression in PMA-induced megakaryocytes at 4 and 7 days of differentiation. The relative mRNA expression level was plotted against gene expression level for the K562 cells. Data are represented as mean ± standard deviation of three independent experiments. MK, megakaryocytes; *, $p < 0.05$ versus K562

**Table S1.** Primer sequences for qPCR

| Name | Product | | Primer sequences (5′ to 3′) |
|---|---|---|---|
| *A2M* | 284 bp | F | TGCTGTACGGTAAAACTCCTTCT |
| | | R | GAGTACGTCATTCTCCGCCT |
| *c-KIT* | 119 bp | F | ATTCAAGCACAATGGCACGG |
| | | R | ACAGGGTGTGGGGATGGATT |
| *CCL2* | 244 bp | F | AGCCACCTTCATTCCCCAAG |
| | | R | TTGGGTTTGCTTGTCCAGGT |
| *CCR4* | 288 bp | F | TTGCCTCACAGACCTTCCTC |
| | | R | AACAGGACCAGAACCACCAC |
| *CD34* | 176 bp | F | AGACTGTGCAGTGATGTGGT |
| | | R | CCCTGGTACATT CGGGTCTG |
| *CD41* | 148 bp | F | AGGCCTCTGGATCAGTTTGTG |
| | | R | TTGGAATGGCCCTCTCCTCCA |
| *CD44* | 105 bp | F | GCTGCAGCCTCAGCTCATA |
| | | R | CATGGGGTGTGAGATTGGGT |
| *CGA* | 146 bp | F | TCAACCGCCCTGAACACATC |
| | | R | TCTGGGCAATCCTGCACATC |
| *CILP* | 223 bp | F | TTTCTTGGCCTTGCTCCACA |
| | | R | ACCAGGAGATGTTCTTTCCAGAC |
| *COL6A5* | 256 bp | F | CTGAGACAGCTACTGATTTGATGC |
| | | R | CCTGTCATTTACCACAGTACCTCT |
| *FN1* | 220 bp | F | AGGAAAAAGACAGGACAAGAAGC |
| | | R | GCCTCTGCTGGTCTTTCAGT |
| *GATA1* | 155 bp | F | TGGTGGCTTTATGGTGGTGG |
| | | R | GGAAAGGCATGAGGTGGCTA |
| *GATA2* | 154 bp | F | GCGAAGACTGACGACAACCA |
| | | R | GGACATCTTCCGGTTCCGAG |
| *GP1B* | 262 bp | F | TTCCTTCGAGGTTCGCTTCC |
| | | R | CACCCTGTGTTCCTTACCCC |
| *GPR4* | 166 bp | F | CCGTTGTCAAGACCGGGGA |
| | | R | AATTTCAATGGGGGCAGGGG |
| *HEY1* | 166 bp | F | GGCTGGTACCCAGTGCTTTT |
| | | R | TCCCGAAATCCCAAACTCCG |
| *IL1A* | 157 bp | F | GGCGTTTGAGTCAGCAAAGAA |
| | | R | GGAGTGGGCCATAGCTTACA |
| *IL5RA* | 177 bp | F | CGCTTATCTGTAAAATAAAGCTGGG |
| | | R | GAGCCAGCATCCCTGTTCTT |
| *IL20RB* | 300 bp | F | GAAAGATGGCTGAGATGGACAGA |
| | | R | TCTCGTACTCCCCCTGGTATT |
| *ITGA9* | 267 bp | F | CCCCACTGGAGAAGGGTAGA |
| | | R | TAACTCCCCAGTCCCCGTAG |
| *ITGB3* | 241 bp | F | AGAAGCAGAGTGTGTCACGG |
| | | R | TCCATGGTAGTGGAGGCAGA |
| *LRP12* | 125 bp | F | GGGTGTACGCTTGTGGAGAG |
| | | R | TGGGTTTGCCCTTATGAACCA |
| *LYZ* | 104 bp | F | GGATGGCTACAGGGGAATCAG |
| | | R | TGCTTCTGTCTCCAGCATTGT |
| *MAP1B* | 168 bp | F | CGGAGCGAGACACTTCGC |
| | | R | GACGACCACCAGCAAGTAGAA |
| *PRG2* | 124 bp | F | CCCAGGAAGGTCTCTGGGATA |
| | | R | GCACCCAAAGGGGTCTCAAA |
| *S100P* | 98 bp | F | GAGAAGGAGCTACCAGGCTTC |
| | | R | TCCACCTGGGCATCTCCATT |
| *SERPINF1* | 209 bp | F | AGAGGGACTAGGCTGGGTG |

| | | | |
|---|---|---|---|
| | | R | GCTTGTTCACGGGGACTTTG |
| *SLC18A2* | 288 bp | F | CCATGGCCCTGAGCGAG |
| | | R | GGTCTCTGGTAGCATTCCCG |
| *TCN1* | 116 bp | F | ACTTCTGAGTGGAGGCGAAC |
| | | R | AGCTGAGGAAAGTTTGGGCT |
| *TFPI2* | 120 bp | F | CAGGAGCCAACAGACTACGG |
| | | R | GAAATTGTTGGCGTTGCCCT |
| *VEGFA* | 184 bp | F | TCACCAAGGCCAGCACATAG |
| | | R | GCAACGCGAGTCTGTGTTTT |
| *GAPDH* | 100 bp | F | GCATCTCCCTCACAATTTCCA |
| | | R | GTGCAGCGAACTTTATTGATGG |

*GAPDH*, glyceraldehyde 3-phosphate dehydrogenase; F, forward; R, reverse; Please see abbreviations for the full name of genes.
